# Impact of clinical pharmacist’s interventions on pharmacotherapy management in elderly patients on polypharmacy with mental health problems including quality of life: A prospective non-randomized study

**DOI:** 10.1038/s41598-019-53057-w

**Published:** 2019-11-14

**Authors:** Matej Stuhec, Nika Bratović, Aleš Mrhar

**Affiliations:** 1 Department of Clinical Pharmacy, Ormoz Psychiatric Hospital, Ptujska cesta 33, SI-2270 Ormoz, European Union Slovenia; 2 University of Ljubljana, Faculty of Pharmacy, Aškerčeva cesta 7, SI-1000 Ljubljana, European Union Slovenia

**Keywords:** Geriatrics, Outcomes research

## Abstract

Mental health problems (MHPs) are very common in the elderly and can have an important influence on their quality of life (QoL). There is almost no data on the impact of clinical pharmacists’ (CPs) interventions on the QoL including elderly patients and MHPs. The main aim of this study was to determinate the impact of (CP’s) interventions on the QoL and quality of pharmacotherapy. A prospective non-randomized pre-post study was designed which included residents of a nursing home aged 65 age or more with at least one MHP. Each patient also filled out the EQ-5D questionnaire. The medical review MR included drug-related problems (DRPs) and potentially drug-drug interactions (pDDIs), as well as potentially inappropriate medications (PIMs). After 2 months, the participants were interviewed again. The mean number of medications before the intervention was 12,2 ± 3,1 per patient and decreased to 10,3 ± 3,0 medications per patient (p < 0,05) (n = 24). The total number of PIMs and pDDIs was also reduced and QoL was also significantly higher (p < 0,05). A collaborative care approach with a CP led to a decrease of DRPs, pDDIs, PIMs, the total number of medications and to an improvement in the patients’ QoL.

## Introduction

The population in Europe is aging, which is increasing drug consumption and the frequency of polypharmacy, creating a need for interventions optimizing pharmacotherapy. In this context, psychotropics are the most important, especially because their use can lead to many important drug-drug interactions (DDIs) and adverse drug reactions (ADRs). This can lead to treatment failure and increased care costs. A 2006 study of elderly residents in Slovenian nursing homes reports that 73% of the participants were treated with at least one psychotropic drug, which was comparable to other European countries^1^. The most commonly used drugs in this study were hypnotics and sedatives. The concurrent use of several different psychotropics (e.g. antipsychotic polypharmacy, APP) was also common, though there is little evidence for the treatment efficacy and safety of APP^1^. Psychotropics are primarily used to treat mental and behavioral disorders, which means that a diagnosis is needed for prescribing psychotropics^1–3^. Although controlled trials are valuable, they can often exclude patients with polypharmacy in pursuit of experimental validity, so there is a need for well-designed studies with strong ecological validity in clinical practice in order to improve the management of polypharmacy.

Several medication lists can help to ensure prudent psychotropic prescribing in elderly patients. Some of the psychotropic drugs are listed on internationally recognized lists of potentially inappropriate medications (PIMs) in the elderly such as the PRISCUS list, the Beers criteria and the STOPP/START criteria^3–5^, which means that effective tools are available in clinical practice to minimize PIMs. Another Slovenian study reports that 22.3% of the elderly in the nursing home had at least one PIM according to the Beers criteria, in most cases a psychotropics drug^6^. In addition, an extensive national Austrian retrospective study found that 55% of all prescribed PIMs included psychotropic drugs in nursing homes^7^. The most frequent PIMs were antipsychotics, especially low-potency antipsychotics in low doses (as a treatment for insomnia and restlessness). In another study investigating the prevalence of PIMs in elderly patients admitted to a psychiatric hospital, at least 1 PIM was reported in 79% of the patients, of which 70% were psychotropics^8^.

PIMs prescribing in elderly patients can cause harm and result in treatment failure or increased treatment costs^9–13^. Lau *et al*. showed that patients with PIMs in therapy had a higher likelihood of hospitalization due to PIM treatment over a one-year period (odds ratio (OR) = 1.73; 95% confidence interval (CI) 1.14–2.60) and higher mortality (OR = 1.89; 95% CI 1.47–2.44), which increased treatment costs^9^. A higher risk for hospitalization (OR = 1.80, 95% CI 1.34–2.41) and higher mortality (OR = 1.28, 95% CI 1.05–1.55) were also observed in nursing home residents exposed to PIMs for at least two months^9^.

A collaborative care approach is one way to limit the risks of PIMs and DDIs. A collaborative care approach including a clinical pharmacist (CP) in patients with mental health problems (MHPs) has been described in some papers outside Europe^14,15^. For most European countries, there is no data on the potential impacts of CPs’ interventions on the pharmacotherapy optimization process in elderly patients with MHPs and polypharmacy, therefore additional prospective studies rooted in clinical practice are needed to examine the merits of collaborative care. The Slovenian Pharmacy Act defines the goal of a medical review (MR) to be the improvement and maintenance of the patient’s health-related quality of life (QoL)^16^. However, there are very few prospective design studies investigating the impact of CPs’ interventions on the QoL in elderly patients with MHPs and polypharmacy. The meta-analysis by Huiskes *et al*. examines the effectiveness of drug reviews on various treatment outcomes, but found no impact on the patients’ QoL^17^. The meta-analysis only included 8 studies, so further research on the relation between CPs’ interventions and QoL measures is needed. In addition, previous studies in Slovenia have shown that elderly patients with MHPs are often treated with important PIMs and DDIs, so this pharmaceutical service could be an important tool for pharmacotherapy management in this population^1,16^.In most European countries there is no data on the impact of CPs’ interventions on the QoL and pharmacotherapy quality in elderly patients with MHPs and polypharmacy, so the main aim of this study is to address this lack of research.

## Methods

### Study design and data collection

A prospective non-randomized pre-post study was carried out in 2017 in one Slovenian nursing home in SW Slovenia with approximately 200 residents. In this prospective pre-post study two main study points were defined: 1) the first patient interview (including an EQ-5D assessment) and MR preparation, and 2) monitoring after 2 months. A general practitioner (GP) referred patients to participate in the study according to the inclusion criteria (65 age or more, at least one mental health problem, at least one psychotropic drug, written consent, cognitive and intellectual ability to fill out the EQ-5D questionnaire). The criteria for the presence of a mental health problem was a mental or behavioral diagnosis according to the 10th revision of the International Statistical Classification of Diseases and Related Health Problems (ICD) (ICD-10)^2^. All included patients were screened by a MPharm student (NB) and a psychiatric CP (MS), who approved all final versions of the MRs, which were sent to the GP. The GP then made a final decision which interventions would be accepted. The psychiatric CP (MS) has been working in a psychiatric hospital setting daily for over 8 years; including daily rounding and ward activities and ambulatory clinical pharmacy service.

The initial data acquisition consisted of two phases. The first was the retrieval of the patients’ medical documentation (patients’ medical chart, the records of nursing care and patients’ electronic data) and the second was a patient interview. The data on current pharmacotherapy was based on the medical chart and an electronic medication list provided by the GP (regular and as-needed basis medications were included). Some of the medications that were given to patients without prescription were also included (e.g. bisacodyl, dexapentanol, dextran). Before writing a MR, the CP interviewed the patients individually and obtained data on: hypersensitivity to medications, specialties in the patients’ diet, smoking and excessive alcohol consumption, current and past adverse reactions, dietary supplement use and self-medication. At the end of the interview, patients filled out the EQ-5D Visual analogue scale (VAS) questionnaire to obtain a QoL measure. Patients who were not able to mark the QoL on VAS themselves due to illness showed the score with a finger or gave their answers verbally. Drug adherence was evaluated by a refill-based measure.

A MR was prepared on the basis of medical documentation and patient information within one week after the initial interview (as standardized in the Slovenian Pharmacy Act)^16^. The MR included the following important aspects: potential and clinically important DDIs, possible adverse events, existing drug indications, PIMs and final recommendations depending on the patient’s outcomes. Researchers also examined the adequacy of renal dosing (note the estimate of glomerular filtration indicated on laboratory findings) and the liver function, guidelines, and summary of product characteristics (SmPCs), to examine the dosage regimen. The latest guidelines for the treatment of individual dieseases were considered when preparing the MRs for this study. The German Priscus lists and Beers criteria were used to determine PIMs^18,19^. Potential DDIs (pDDIs) were determined by various interaction classes with Lexicomp® 4.0.1 and 4.0.2 and only X (major interactions which should be avoided) and D (minor interactions to be avoided if possible) were included.

This study only evaluated the effects of collaborative care including a CP and a GP and the cooperation between the GP and the nursing home psychiatrist, who might had been consulted during our study, was not measured. In the nursing home setting of our study, the GP could prescribe medication, but the CP could only propose changes through a MR.

### Impact of the clinical pharmacist’s interventions

The MR was sent to the GP who accepted or rejected the CP’s interventions. NB and MS (the researchers) recorded which interventions the GP accepted in a 2-month period and identified drug-related problems (DRPs). At the end of the 2-month period, the researchers re-examined the medical documentation, recorded the interventions (shown in Table 1) and interviewed the patient again, including the EQ-5D questionnaire. The questionnaire was discussed and resolved as in the first phase of the study (see subsection 3.3). Researchers did not monitor the patients further, as the study ended. Each patient participated in the study only once. The study used the validated Slovenian EQ-5D (EQ-5D-3L) questionnaire (Slovenian version for Slovenia), as confirmed by the EuroQol Research Foundation in 2016.

**Table 1:** Potentially inappropriate medications in the elderly pre- and post-intervention.

	N of cases before review	Final N of cases	Criteria
nitrazepam	6	2	P
methyldigoxin	3	2	P
fluphenazine	1	1	P
solifenacin	1	1	P + B
clozapine	1	1	P + B
doxazosin	1	1	P + B
lorazepam	3	3	P + B
diazepam	3	3	P + B
alprazolam	3	3	P + B
ibuprofen	4	0	B
zolpidem	1	1	B
spironolactone >25 mg daily	1	0	B
risperidone	1	1	B
dabigatran	1	1	B
haloperidol	1	1	B
metoclopramide	1	1	B
Total	32	22	

P – Priscus list, B – Beers criteria, P + B – on both lists.

This study was a non-industry supported study and was approved by the National Medical Ethics Committee of the Republic of Slovenia in 2016 and informed consent was signed by each patient. The methods were carried out in accordance with the relevant guidelines and regulations.

### Drug-related problems

DRPs were classified according to the Slovenian classification of drug-related problems (DRP-SLO-V1) with certain adjustments^20^. DRPs have been identified as potential or expressed. Potential DRPs were described in terms of risk factors, which were potential reasons for the DRP to arise in practice. At the end of the study, we examined whether any of the potential DRPs occurred. The identified problems were identified as problems related to treatment effectiveness, adverse events (treatment safety) and problems associated with unnecessary drug treatment (e.g. no indication).

The following interventions were selected: drug discontinuation, medication initiation, drug regimen adjustment, counseling to correct the inappropriate use of medicines, and monitoring the treatment with disease monitoring if the disease was not treated pharmacologically.

The results of the CP’s interventions were evaluated based on the severity of the problems at the end of the research period, which was evaluated from the information obtained from the patients’ medical documentation at the time of the initial interview and at the end of the study period. Researchers identified the problem as being resolved if they managed to remove the risk factor for the DRP and the patient did not report any problems at the end of the study.

### Statistical analysis

The characteristic of the sample was described using descriptive statistics. The patients were monitored at two time points (at the first interview before the MR and at the end of the study period, after a GP reviewed the MR). The Shapiro-Wilk test was used to test normality. In determining the correlation between individual variables the Pearson correlation coefficient (r) was used. The t-test for the dependent samples (pair t-test) was used for normally distributed variables, and a nonparametric Wilcoxon signed-rank test for non-normally distributed variables. Analyses were carried out with the Statistical Package for Social Science 22.0 for Windows® (SPSS).

## Results

### Clinical pharmacist’s impact on the total number of medications

**Figure 1:**
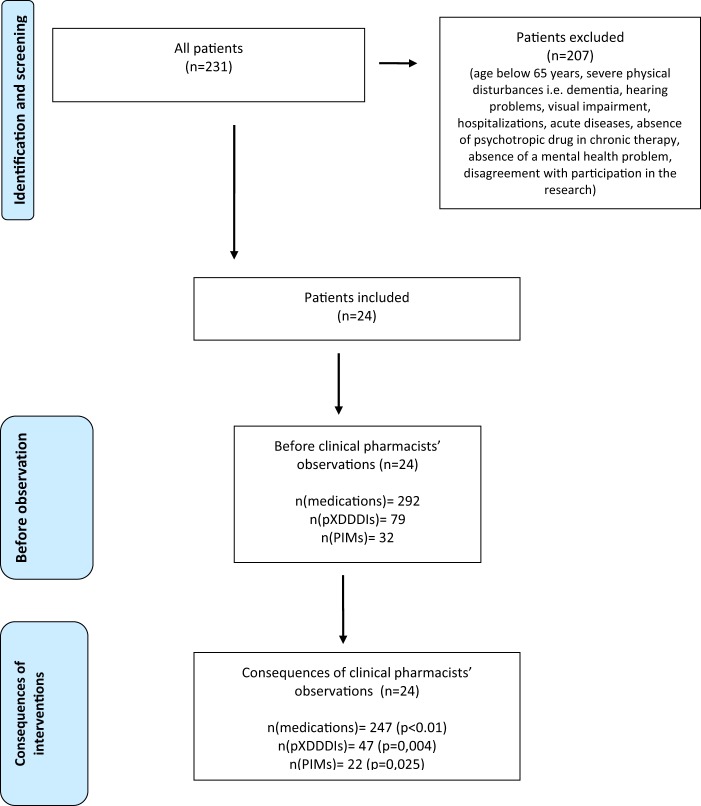
Flowchart.

The study included 24 patients (mean age = 80.6, SD = 6.8; 87.5% women) and 207 patients were excluded from the analysis because of several reasons described in Fig. 1.The mean number of medications per patient before the MR was 12.2 (SD = 3.1) and decreased to 10.3 (SD = 3.0) at the end of the study period (p < 0.05). The highest number of medications per patient was 18 (2 patients) and the lowest 8 (2 patients). In 20 patients (83.3%) the number of medications was reduced (maximum reduction of 5 medications). The number of medications increased only in one patient (1 additional medication).

The Anatomical Therapeutic Chemical (ATC) Classification System was used to classify the medications, with most falling into the group N (35.3%, N = 292), followed by group A (23.3%, N = 292), used for the treatment of gastrointestinal and metabolic diseases, and group C (21.2%, N = 292) for the treatment of cardiovascular diseases. The total number of medications was reduced by 13.6% in the group N (N = 103), 33.3% in group A (N = 68) and 85.7% in group M (N = 7). The number of medications in Group C did not change.

### Potential drug-drug interactions

18 type X pDDIs and 61 type D pDDIs were detected at the start of the study period. There was a positive correlation between the number of group N medications and the number of type X and D pDDIs (r = 0.482, p (bilateral) < 0.05). No type X pDDIs were found in 12 patients (50%, N = 24). The highest number of type X pDDIs per patient was 5. The most common of type X pDDIs was the pDDI between risperidone and ipratropium (4 cases), which was identified as clinically irrelevant.

All pDDIs were identified as potential problems. In addition to the assessment of the medical documentation and patient interview, the researchers also examined which pDDIs were clinically relevant and which interventions were suggested. In 5 patients, the CP proposed an intervention in those pDDIs that could prolong the QT interval and consequently cause syncope, dizziness, irregular heartbeat, dyspnoea, and dizziness, which were not seen during the study period. The CP advised an electromyocardiogram (ECG) and pharmacotherapy substitution in case of QT prolongation. For one participant, the QTc was appropriate (less than 460 ms for women), while for another it was slightly prolonged, but the GP did not decide to change the therapy.

**Figure 2:**
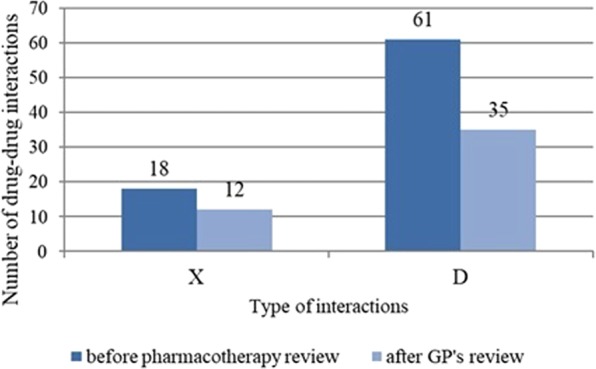
Total number of ATC-classified medications pre- and post-intervention.

At the end of the study period, the total number of type X and D pDDIs had decreased significantly by 33.3% and 42.6% respectively (p = 0.004). Four patients did not have any type D and X pDDIs and in 10 patients, only type D pDDIs were identified at the end of the study (Fig. 2).

### Potentially inappropriate medications in the elderly

No PIMs were identified in 7 patients (29.2%, N = 24). Three patients had PIMs which were only listed on the Priscus list (1 patient) or in the Beers criteria (2 patients). For other patients, medications have been identified that fulfilled both criteria. The highest number of PIMs per patient was 3. 6 medications fulfilled both criteria (Table 1).

In the Priscus list, researchers also found flufenazine, nitrazepam and methyldigoxin, which are suitable for use in the elderly according to Beers criteria. There were 7 medications which were unsuitable only according to the Beers criteria (ibuprofen, zolpidem, haloperidol, metoclopramide, spironolactone at doses higher than 25 mg daily, risperidone in dementia treatment, dabigatran etexilate in patients older than 75 with reduced renal function).

The share of PIMs in all medication taken by the participants was 10.6% (N = 302) at the beginning and 8.4% at the end of the study (N = 262). The reduction of PIMs (defined by the Priscus list) after the intervention was statistically significant (p = 0.025).

### Drug related problems in patients with mental health problems

The total number of DRPs was 165, of which 8% (N = 165) were expressed and the other were identified as potential. The most frequently identified DRPs were related to the treatment of MHPs, followed by the treatment of cardiovascular diseases and pain in patients with MHPs.

DRPs concerning MHP treatment were identified for all of the study participants. Researchers identified two expressed problems and defined them as an ADR related to the use of antipsychotics. For 2 patients, extrapyramidal adverse reactions were expressed. Consequently, the patients received biperidene. Researchers identified all other problems as potential problems (41 problems). The most common risk factor was that the duration of benzodiazepines treatment was too long. This was followed by the use of antipsychotics without approved indication. Only 3 patients had an indication for receiving antipsychotics (schizophrenia and delusional disorders). Other antipsychotics were used for restlessness, behavioral and psychological symptoms in dementia, delirium and insomnia, all without an approved indication for the use of antipsychotics (with the exception of risperidone for behavioral disorders in dementia).

The CP provided 65 separate interventions in this study. The highest proportion of the interventions (46.2%) was a suggestion for drug discontinuation. Medication initiation was suggested in 29.2% interventions, followed by drug adjustment in 13.8% and monitoring in 10.8% of all interventions (Table 2).

**Table 2:** Different intervention types, number of cases and number of accepted interventions within study.

Interventions	Number of cases	Number of accepted interventions
Drug discontinuation	30	11
Medication initiation	19	3
Drug adjustment*	9	4
Treatment monitoring	7	1
Σ	65	19

^*^Lowering dose, elevation of the dose, drug administration, dose frequency.

19 of the 65 proposed interventions were accepted by the GP, which is 29.2% (N = 65). Most of the confirmed interventions were associated with the discontinuation of benzodiazepine treatment. In 6 patients, the CP advised discontinuation and the dose of benzodiazepine was decreased or the dosing regimen was changed from a regular basis to as-needed. A patient reported epistaxis following a change in the dosing regimen of sertraline from 2 × 50 mg to 1 × 100 mg. According to the literature, this cannot be connected to an ADR^21,22^. Another patient who reported a burning sensation in the stomach after using venlafaxine and galantamine and was advised to take medication at a meal, was still reporting problems at the end of the study. In one case, the GP excluded diagnosis of Parkinson’s disease during research so the GP could not take into account the CP’s proposals for the treatment of Parkinson’s disease. The GP consulted a psychiatrist in 4 patients. The psychiatrist discontinued olanzapine in one patient and other interventions were not accepted by GP. With the intervention the CP managed to reduce the number of risk factors by 16 (29.1%, N = 55).

### Quality of life measures

**Figure 3:**
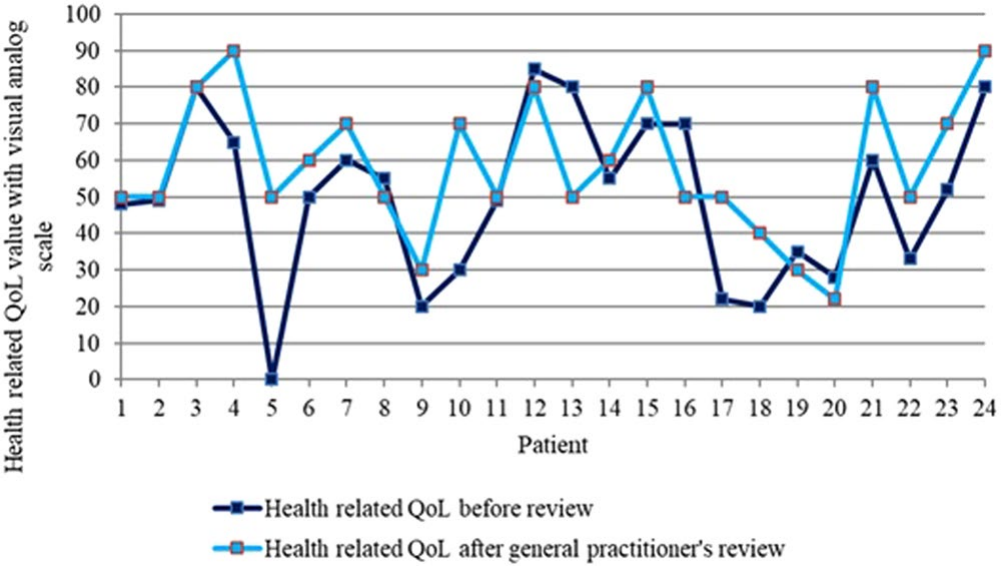
Clinical pharmacist’s impact on health-related quality of life during the study.

The health-related QoL using the VAS was significantly higher at the end of the study (p < 0.05). Increases in the QoL measure were observed in 17 patients (70.8%, N = 24; Fig. 3). The biggest change was an increase in 50 points (from 0 at the first and 50 at the second measurement). The greatest reduction in health-related QoL was 30 units (from 80 to 50).

The most important improvement in the descriptive system was achieved in the anxiety and depression part. 18 patients (75%, N = 24) were moderately or extremely anxious or depressed at the first QoL measurement and that number decreased to 14 (58.3%, N = 24) patients at the end of the study. Only 1 patient was extremely anxious or depressed at the end of the study. The degree of pain changed in 6 patients (25%, N = 24) by the end of the study period. 3 patients (12.5%, N = 24) reported a decrease of pain after at the end of the study period. The Pearson’s correlation coefficient between polypharmacy reductions and change in QoL indicated the absence of correlation (p = 0.336, r = 0.205).

## Discussion

This was the first prospective study assessing the impact of a pharmacist-focused collaborative care approach on the QoL of elderly patients with MHPs in Central Europe. The promising results open a new window into treatment optimization and QoL elevation resulting from involving a CP in the treatment process. This study corroborates two US studies showing that pharmacist-focused collaborative care improved the outcomes of patients with depression^14,15^. It also shows that similar benefits can be achieved for elderly patients with MHPs and polypharmacy, especially involving psychotropics.

The first important outcome of this study is that the CPs involvement reduced the the number of medications per patient, which reduces the risk of irrational polypharmacy and subsequent DRPs. These results corroborate previous studies showing that CPs’ interventions reduced the total number of medications^23,24^. One strength of our study is also that the interventions achieved positive results for patients with MHPs concurrently treated with a relatively large number of medications.

The second important result is the reduction of total pDDIs after the CP’s interventions, which can lower mortality and morbidity^25–27^. The number of clinically important DDIs was also reduced. The most frequent pDDIs were related to antidepressants and antipsychotics and the CP suggested alternatives in these cases. These results show that cooperation between CPs and GPs can facilitate optimal pharmacotherapy for elderly patients with MHPs and polypharmacy, as well as be a powerful approach to reducing pDDIs in general, although further research with larger samples and measures of long-term clinical outcomes is still needed.

The third important outcome is that the CP’s interventions also reduced the number of PIMs and provided appropriate alternatives. Almost 75% of the patients were treated with at least one PIM. Benzodiazepines were often discontinued, in line with the guidelines^18^. Ibuprofen was also often discontinued and paracetamol suggested as a substitution, in line with clinical guidelines. 75% of PIMs were related to psychotropics, corroborating an Austrian study finding psychotropic drugs accounted for 55% of the PIMs^7^. Overall, the results are similar to those by Milos and *et al*. and Stuhec *et al*., reporting a dramatic reduction of the number of PIMs in the elderly in primary care following a CP’s interventions^28,29^. DRPs were reduced by the interventions as well. The CP’s impact was highest on drug discontinuation (46.2%) followed by drug initiation, which was suggested in 29.2% of the cases. A US study reports only 7% of the CPs’ interventions were related to drug discontinuation^24^, suggesting medications in our study were frequently used without approved indication and were reduced after the CP’s interventions.

Our study was also the first in Central Europe to use QoL measures to assess the impact of the CP’s interventions in the elderly with MHPs treated with polypharmacy, which revealed a positive effect in contrast with a 2017 meta-analysis, though replication is desirable due to a small sample size^17^.

Lastly, many treatment guidelines inconsistencies have been discovered for all groups of psychotropics. In 13 patients, benzodiazepines were taken for several years despite their association with ADRs, especially falls. The GP accepted most of the CP’s recommendations related to psychotropics. Some patients were also treated with APP and two patients were treated with APP without prior clozapine monotherapy, which goes against the recommended guidelines. Both patients have also been treated with biperiden, not recommended in elderly patients because its anticholinergic properties^5^, and had ADRs (extrapyramidal symptoms) detected by the CP.

Additionally, a guideline-approved indication for antipsychotic treatment^30,31^ was found in only 3 out of 18 patients treated with antipsychotics. The CP suggested appropriate interventions in these cases, which were mostly rejected by the GP, despite guideline recommendations against the frequent use of antipsychotics in the elderly with a MHP, with the DART-AD trial results especially stressing discontinuation in dementia patients^30,31^. The use of antipsychotics to treat the behavioral symptoms of dementia has also been reported to be associated with greater mortality. The crude 6-month mortality rates were as follows: haloperidol 20.0%; olanzapine 12.6%; risperidone 12.5%; valproic acid and its derivatives 9.8%; and quetiapine 8.8% (p < 0.0001)^31^. These results show that the CP in our study suggested important antipsychotic-related interventions to reduce mortality, although they were mainly not accepted by the GP. The CP recommended substituting frequent small doses of quetiapine with the antidepressant mirtazapine in small doses (e.g. 15 mg daily) and trazodone, which have more favorable interactions profile than quetiapine, which is not recommended as a first-line treatment for insomnia^32^. In the case of antidepressants, the CP mainly advised antidepressant substitution in cases of chronic pain, insomnia and Parkinson’s disease, but the changes were confirmed by the GP in only one case, in which escitalopram and nitrazepam were replaced with mirtazapine and the patient reported improved sleep quality and reduced restlessness. Overall, these results show that the CP played an important role in optimizing antipsychotics prescribing, which elevated clinical outcomes in terms of QoL measures. The results also corroborate an Austrian study that reports antipsychotics are frequent in PIMs^7^. Our and the Austrian study point to a need for changes in antipsychotic prescribing practice so that antipsychotics are not used in elderly patients with MHPs without a clear indication.

Our study has several limitations. Patients for this study were selected by the GP on the basis of inclusion criteria without a collaboration of a psychiatrist, which may be a source of selection bias. Additionally, only patients without missing data needed for the CP’s medical review were included. The study is also limited by the QoL measure used, the absence of long-term outcome measures, no monitoring between the study points, and its prospective design (i.e. absence of a control group, a non-randomized trial), as well as the the heterogeneity of the patients’ diagnoses and a small sample size. An additional limitation is due to the small number of included participants in the analysis, which can be explained by the high selectivity of the inclusion criteria. Many patients were unable to enter the study due to several mental and physical disturbances. Another important limitation is the lack of direct collaboration between the clinical pharmacist, psychiatrist and GP, which could have led to the low number of accepted interventions. Further studies could examine how GPs decide to accept or reject the proposed interventions. Lastly, the CP did not have prescribing rights and only provided a medical review which was sent to the GP, who decided to accept or reject the recommendations. After two months, the CP only documented all medications again and remeasured the QoL (determining the pre post difference). We assume that further studies could examine how GPs decide to accept or reject the proposed interventions. In spite of these limitations, this study still offers new insights into the merits of a pharmacist-focused collaborative care approach and expands the knowledge on collaborative model implementation in Central Europe.

## Conclusion

A pharmacist-focused collaborative care approach led to a decrease of DRPs, pDDIs, PIMs and increased positive clinical outcomes in terms of QoL measures. This study could serve for future research on pharmacist-focused collaborative care in primary care settings in elderly patients with MHPs in Central Europe. Despite promising results, there is a need for further research employing a randomized or quasi-randomized controlled design to better evaluate the CPs impact on this population.
